# Transungual excision of a large subungual glomus tumor

**DOI:** 10.1016/j.jdcr.2025.12.004

**Published:** 2025-12-11

**Authors:** Ma. Veronica Pia N. Arevalo, Val Constantine S. Cua

**Affiliations:** Department of Dermatology, University of the Philippines – Philippine General Hospital, Manila, Philippines

**Keywords:** glomus tumor, nail matrix tumor, pain, subungual region, transungual excision

## Introduction

Subungual glomus tumors are rare, benign neoplasms arising from the glomus body, a specialized neuromyoarterial anastomosis involved in thermoregulation.^1^ Patients often report reddish purple nail discoloration, pinpoint tenderness, cold sensitivity, and severe paroxysmal pain. Because of the rarity and often subtle clinical presentation, diagnosis can be delayed or missed, leading to prolonged patient discomfort and mismanagement. We describe the case of a middle-aged woman who did not present with the classic triad of a subungual glomus tumor. This case emphasizes diagnostic pitfalls and highlights the value of surgical excision in both diagnosis and cure.

## Case report

A 50-year-old woman consulted for a 5-year history of intermittent sharp pain and point tenderness on the proximal nail fold of the left thumb, associated with gradual nail overcurvature. She denied cold sensitivity, history of trauma, or manipulation of the affected digit. Previously, two physicians had diagnosed her with onychomycosis, treated unsuccessfully with antifungals.

**Fig 1:**
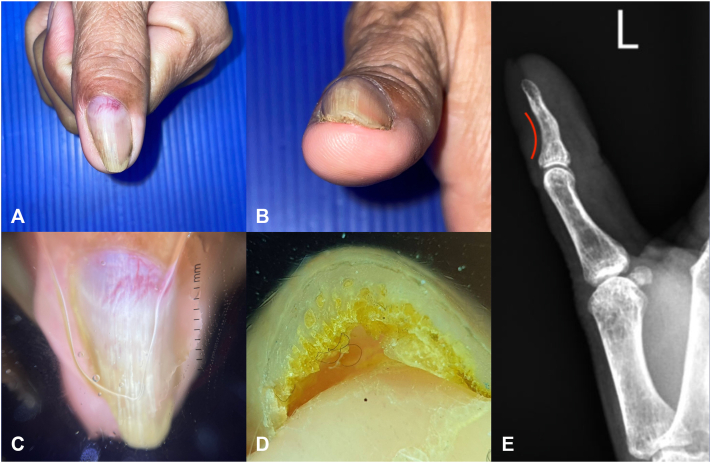
Clinical, dermoscopic, and radiographic images. **A,B,** The left thumbnail has distal onycholysis with an overcurvature on one side. **C,** Dermoscopy shows a structureless purplish area with prominent vasculature overlying the area of the lunula and longitudinal parallel white lines starting from the border of the lunula going toward the distal edge of the nail plate. **D,** Honeycomb-like cavitations are visualized on the free edge of the nail plate. **E,** X-ray views of the left hand showed scalloping (*red curve*) of the outer cortical margin of the dorsal distal phalanx of the thumb with a surrounding ill-defined soft tissue density with no internal calcification.

Examination revealed an overcurved, thickened nail plate with purplish discoloration and prominent vasculature (Fig 1, *A, B*). Distal onycholysis, longitudinal white and yellow bands, subungual hyperkeratosis, and honeycomb-like cavitations on the free edge were also present (Fig 1, *C, D*). Potassium hydroxide mount of nail clippings was negative for hyphal elements. X-ray showed scalloping of the distal phalanx (Fig 1, *E*). Focused ultrasound with color flow study revealed a vascular hypoechoic mass beneath the nail bed.

**Fig 2:**
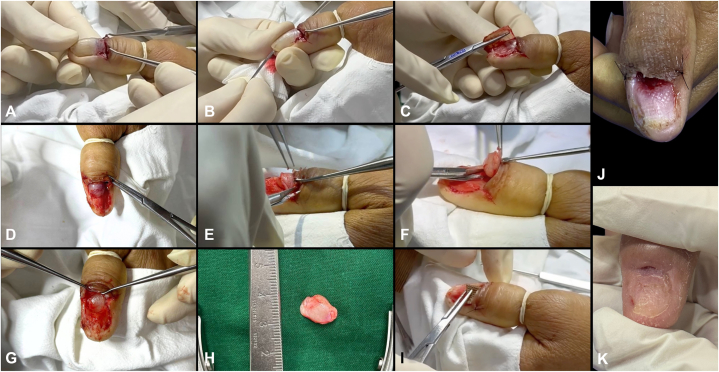
Surgical technique and postoperative care. **A,** Lateral incisions were made on the proximal nail fold, and skin hooks were used to retract the proximal nail fold. **B,** A nail elevator was used to separate the nail plate from the nail bed. **C,** Lateral nail plate curl technique was used for total avulsion. **D,** A well-encapsulated mass was visualized on the proximal nail bed and matrix. **E,F,** The tumor was carefully excised after identifying a plane of dissection. **G,** A large defect was visible after excision, with the distal phalanx palpable underneath. **H,** The well-encapsulated, fleshy, round tumor measured 0.9 × 0.8 × 0.5 cm. **I,** A lipidocolloid silver dressing was inserted in the proximal nail-fold cavity to ensure a moist wound healing environment that can assist with wound bed granulation. **J,** One week after surgery, there was noted good wound bed granulation on the large cavity between the proximal nail fold and nail matrix after excision of the tumor. **K,** Decrease in size of cavity with weekly wound care using lipidocolloid silver dressing secured with self-adherent cohesive bandage.

Based on the clinical findings, a nail matrix tumor was suspected. Total nail avulsion was performed (Fig 2). Intraoperatively, a well-encapsulated, fleshy, round tumor (0.9 × 0.8 × 0.5 cm) was seen invading the nail matrix and abutting the distal phalanx. A dissection plane was identified, facilitating enucleation of the tumor with the capsule intact. After surgery, a lipidocolloid silver dressing was inserted between the proximal nail fold and the operated site and secured with a self-adherent cohesive bandage.

**Fig 3:**
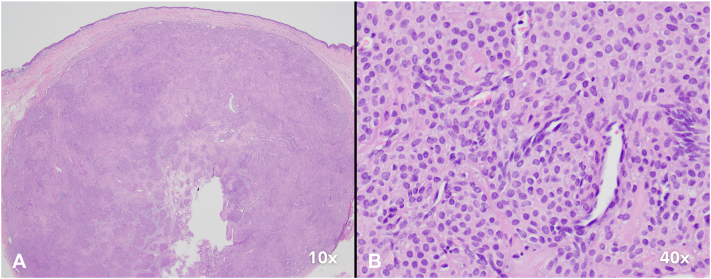
Histopathologic findings of a glomus tumor. **A,** Low-power view shows a well-circumscribed dermal tumor. **B,** High-power view shows that the tumor is composed of monomorphic, round cells with eosinophilic cytoplasm, surrounded by a few dilated blood vessels. (**A** and **B;** original magnifications: **A,** ×10; **B,** ×40.)

Histopathology of the tumor showed a well-circumscribed, dermal nodular proliferation of small, monomorphous cells with round, central nuclei and eosinophilic cytoplasm around dilated blood vessels, consistent with a glomus tumor (Fig 3). Nail plate sections showed changes of candidal onychomycosis.

The patient followed up weekly for wound inspection and dressing change. The surgical cavity progressively granulated and shrank, with resolution of pain and no residual paresthesia after 1 month.

## Discussion

Glomus tumors arise from the glomus body or the Sucquet-Hoyer canal, an arteriovenous shunt surrounded by modified smooth muscle cells.^1^^,^^2^ Majority are located subungually, given the high concentration of glomus cells in the fingertips. The classic triad of paroxysmal pain, localized tenderness, and cold sensitivity characterizes glomus tumors but is not always present, as illustrated in this case by the absence of cold sensitivity.^1^, ^2^, ^3^, ^4^ Pink to purple discoloration may also be seen.^1^^,^^2^^,^^4^ Subungual glomus tumors are more common in middle-aged women and typically show a 4- to 7-year diagnostic delay.^2^^,^^3^^,^^5^, ^6^, ^7^

A positive cold sensitivity test, Love's pin test indicating pinpoint tenderness, and Hildreth's sign—reduced pain following exsanguination with a tourniquet—support the diagnosis.^2^^,^^5^ Although the Love's pin test is useful, its specificity may be limited in larger lesions, such as in this case, where mass effect from large nail unit tumors can cause pain regardless of etiology. Hildreth's sign was not tested initially, as a glomus tumor was not considered at first because of its rarity.

Important differentials include onychomatricoma, superficial acral fibromyxoma, and onychomycosis, although these are generally painless and have distinct nail findings. Although honeycomb cavities in this case may be suggestive of onychomatricoma, the overall presentation is still consistent with a glomus tumor. Painful digital tumors warrant considerations such as neuroma, leiomyoma, mucous cyst, exostosis, and malignancy.^6^

Revisiting nail anatomy can help localize the tumor and guide the surgical approach.^6^ In this patient, dorsal and ventral nail plate changes suggest a matrix origin involving both proximal and distal areas. Discoloration and overcurvature point to a nail bed pathology,^6^ which is likely secondary involvement here due to the large tumor size and the tumor vascularity.

**Fig 4:**
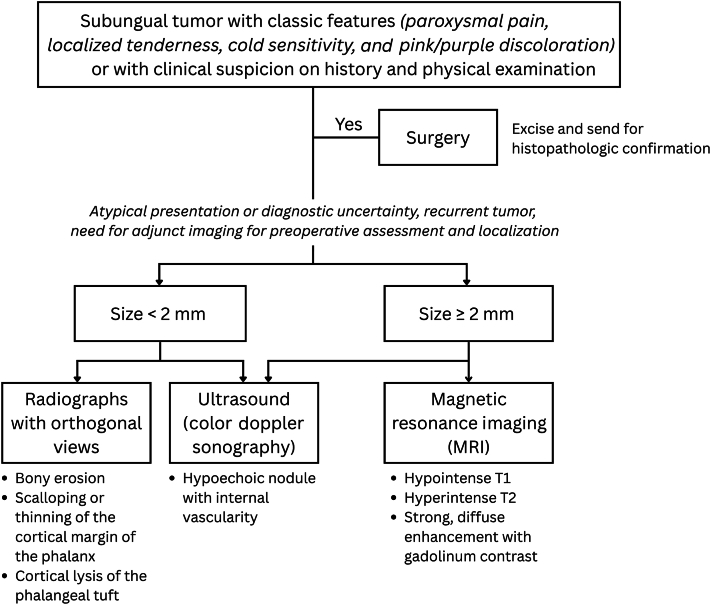
Suggested diagnostic approach to subungual glomus tumors.

Doppler ultrasonography is often the first-line imaging modality because it is inexpensive and widely available, with a high detection rate for glomus tumors as small as 2 mm in diameter.^1^^,^^2^ In contrast, magnetic resonance imaging is regarded by some as the most useful and accurate for preoperative localization and tumor size assessment,^8^ but its high cost and low specificity hinder its utility compared to ultrasound.^1^^,^^2^^,^^9^ Radiographs are less sensitive but can reveal adjunct findings (Fig 4).^1^^,^^2^^,^^5^ It can also help exclude painful bone-related conditions (e.g., subungual exostosis, osteochondroma), complementing ultrasound in initial imaging.^6^^,^^9^ Bony changes may also guide deeper resection up to the bone to prevent recurrence.^6^ In Fig 4, we present a simplified diagnostic flowchart for subungual glomus tumors, highlighting that imaging is supportive but not essential before surgical excision, which remains both diagnostic and therapeutic.

Surgical excision is curative but may be challenging for nail matrix tumors.^1^^,^^4^, ^5^, ^6^ This can be done via different approaches, and the transungual approach offers good visualization with low risk of postoperative nail deformity if the nail is preserved.^2^^,^^4^^,^^7^^,^^9^ In this case, the deformed nail was sent for histopathologic assessment and could not be returned. Unfortunately, the patient was lost to follow-up, preventing long-term outcome assessment. Although tumor recurrences are often associated with incomplete excision,^2^^,^^4^^,^^6^ intrinsic tumor characteristics, such as gene expression and histologic type, may also influence recurrence rate despite appropriate surgical technique.^3^ When nail preservation is not possible, meticulous wound care with appropriate dressings supports recovery and optimizes outcomes.

This case adds to existing literature by illustrating an unusually large subungual glomus tumor with extensive clinical and intraoperative documentation. Beyond the rarity of presentation, this report offers practical and educational value by detailing a dermatologist-performed transungual excision and presenting a practical diagnostic approach for subungual glomus tumors. We emphasize the importance of meticulous documentation and underscore that comprehensive management of nail unit tumors lies well within the scope of dermatologic practice.

## Conflicts of interest

None disclosed.
